# ABL1 Is a Prognostic Marker and Associated with Immune Infiltration in Hepatocellular Carcinoma

**DOI:** 10.1155/2021/1379706

**Published:** 2021-08-26

**Authors:** Rongqiang Liu, Weihao Kong, ZeKun Jiang, Shiyang Zheng, Xiaofeng Yuan, Linsen Ye

**Affiliations:** ^1^Department of Hepatic Surgery and Liver Transplantation Center, The Third Affiliated Hospital of Sun Yat-Sen University, Guangzhou 510630, China; ^2^Department of Hepatobiliary Surgery, The First Affiliated Hospital of Guangzhou Medical University, Guangzhou 510220, Guangdong, China; ^3^Department of Emergency Surgery, The First Affiliated Hospital of Anhui Medical University, Hefei, Anhui 230022, China; ^4^Department of Gastrointestinal Surgery, The First Affiliated Hospital of Guangzhou Medical University, Guangzhou 510220, Guangdong, China; ^5^Department of Breast Surgery, The Third Affiliated Hospital of Guangzhou Medical University, Guangzhou 510150, China; ^6^Department of General ICU, The Third Affiliated Hospital of Sun Yat-Sen University-LingNan Hospital, Guangzhou 510630, China

## Abstract

**Background:**

The role of ABL1 in hepatocellular carcinoma (HCC) is still unclear. Therefore, this study aims to explore the potential role of ABL1 in the progression of HCC using bioinformatics methods.

**Methods:**

We analyzed the expression, prognostic potential, and immune cell effect of ABL1 in HCC by using a variety of datasets.

**Results:**

ABL1 is highly expressed in HCC and associated with unfavorable overall survival (OS) and disease-free survival (DFS). Functional network analysis revealed that ABL1 plays an important role in mitochondrial activity, ATP metabolism, protein translation and metabolism, various neurological diseases, nonalcoholic fatty liver disease, and notch signaling pathway. In addition, we found that ABL1 expression was closely correlated with B cells, CD8 + T cells, CD4 + T cells, macrophages, neutrophils, and dendritic cells. Furthermore, ABL1 expression was positively associated with the expression levels of immune checkpoint genes, such as PD-1L, TIM3, TIGIT, and CTLA4.

**Conclusion:**

ABL1 is associated with immune infiltration and prognosis of HCC.

## 1. Introduction

Hepatocellular carcinoma (HCC) is the most common primary liver tumor and the second most common risk factor for cancer death worldwide [1]. In China, approximately 400,000 people die from liver cancer each year, accounting for more than 50% of the global liver cancer death rate [2]. According to statistics, the incidence of liver cancer will continue to rise in the next 20 years [3]. HCC prevention and treatment have become an urgent problem for the global medical community. In its early stages, HCC is occult and difficult to detect; thus, patients with HCC are often diagnosed when the cancer has already advanced or metastasized. Although great progress has been made in the comprehensive clinical treatment of HCC, the prognosis of patients with advanced liver cancer is still poor [4]. Therefore, finding new and specific tumor markers to effectively predict the prognosis of HCC patients is of great value for guiding clinical treatment and improving patient survival.

The human Abelson tyrosine-protein kinase 1 (ABL1) gene is located on chromosome 9q34, and the ABL1 protein encoded by it belongs to the ABL interacting protein family [5]. ABL1 plays a role in a wide range of normal biological functions, including cytoskeleton remodeling [6]. In 1998, Taki et al. revealed that ABL1 was associated with the occurrence of chronic myeloid leukemia [5]. Since then, knowledge of the roles of ABL1 has gradually increased. Subsequent studies have shown that ABL1 is abnormally expressed in a variety of tumors and plays a crucial role in tumor proliferation, migration, invasion, and metastasis [7]. ABL1 regulates a variety of signaling pathways, such as the EGF and PI3K/AKT signaling pathways [8, 9]. In addition, ABL1 has been found to be closely associated with the prognosis of some tumors, including gastric and breast cancers [10, 11].

So far, only a few studies have reported the relationship between ABL1 and HCC. Chitsike et al. indicated that ABL1 was abnormally expressed in human liver cancer [12]. Wang et al. further found that overexpression of ABL1 promoted tumor progression through NOTCH1 in mouse liver cancer [13]. However, the prognosis and clinical value of ABL1 in HCC in humans is still unclear.

Immune infiltration is known to be closely related to the progression of liver cancer [14]. In this study, we used a variety of databases to comprehensively explore the clinical significance of ABL1 in liver cancer, identify possible target pathways, and determine the relationship between ABL1 and immune infiltration. Our results provide a better understanding of the potential value of ABL1 in liver cancer.

## 2. Materials and Methods

### 2.1. Data Acquisition

Datasets with gene expression profiles and clinical information regarding HCC mRNA expression were downloaded from the TCGA database [15] (https://TCGA data.nci.nih.gov/tcga/). Standardization was performed on the downloaded HCC datasets, and cases that did not contain survival information were excluded. The total number of samples in the original study of the TCGA data was 529, consisting of 369 liver cancer tissue samples and 160 paracancerous tissue samples. Five HCC samples contained no survival information. Using the median of ABL1 mRNA expression as a node, HCC tissue samples with survival information were divided according to ABL1 expression levels into a high expression group and a low expression group, with 182 samples in each group.

### 2.2. Survival and Expression Analyses

The online database Gene Expression Profiling Interactive Analysis (GEPIA) [16] was used to analyze the expression of ABL1 in HCC tissues and normal tissues and further assess the prognostic value of ABL1 in liver cancer. In addition, we conducted univariate or multivariate analysis on the clinicopathological information available on ABL1 in HCC. Moreover, we used the online database UALCAN [17] to evaluate the expression of ABL1 protein in different groups.

### 2.3. Functional Enrichment Analysis

The LinkedOmics database [18] is a free public website that can analyze TCGA data. The LinkedOmics database was used to analyze ABL1-related genes. The Pearson correlation coefficient was applied for statistical analysis, and the results are expressed as a volcano map and a heat map. In addition, Gene Ontology Biological Process (GOBP) and Kyoto Encyclopedia of Genes and Genomes (KEGG) pathway analyses were performed. Genes with a discovery rate (FDR) < 0.05 were considered to be significantly enriched.

### 2.4. TIMER Database Analysis

TIMER [19] is a visualization website that can perform automatic analysis and correlate the immune penetration level and immunogenicity. The TIMER website uses deconvolution to analyze gene expression profiles to infer the expression of tumor-infiltrating immune cells. We used the TIMER database to explore the relationship between the expression abundance of various immune cells and the expression of ABL1 in HCC.

### 2.5. Evaluation of Tumor Microenvironment via CIBERSORT

CIBERSORT [20] can assess changes in the expression of specific genes in tissue based on a deconvolution algorithm. We used CIBERSORT to evaluate the immune response of 22 immune cell types in HCC. The “Vioplot” package was used to visualize the differences in the 22 TIICs between the high and low ABL1 expression groups. In addition, TISIDB [21] was used to analyze the relationship between ABL1 expression and 28 types of tumor-infiltrating lymphocytes.

### 2.6. Statistical Analysis

The statistical data obtained from TCGA were processed by *R* 3.5.3, and the *P* value < 0.05 was considered statistically significant. The survival rate was analyzed by a log-rank test and Mantel–Cox test. Logistic regression was used to analyze the correlation between clinical features and ABL1 expression. The correlation between the expression of ABL1, coexpressed genes, and 22 immune cell types was measured by the Pearson correlation coefficient.

## 3. Results

### 3.1. Expression Levels and Prognostic Value of ABL1 in HCC

**Figure 1:**
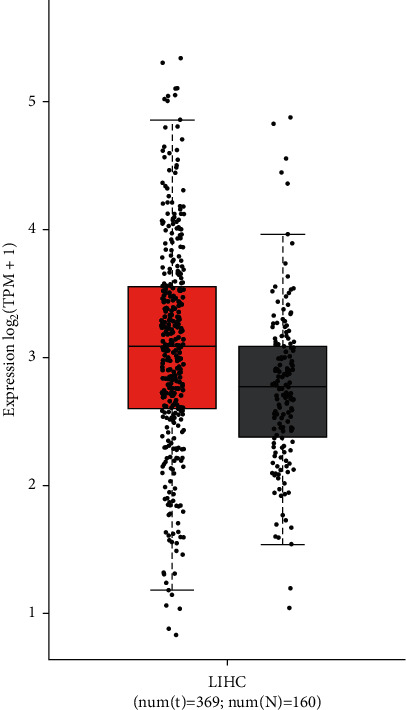
ABL1 mRNA expression in HCC. ABL1 mRNA expression in liver cancer tissues is significantly higher than that in normal tissues.

**Figure 2:**
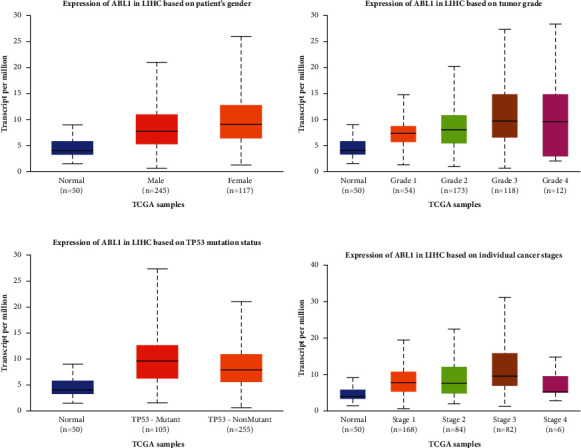
ABL1 protein expression varies in various subgroups of HCC patients based on patient age, sex, cancer stage, and TP53 mutation status. (a) Box plot shows the relative expression of ABL1 in different age groups of HCC patients. (b) Box plot shows the relative expression of ABL1 in different sex groups of HCC patients. (c) Box plot shows the relative expression of ABL1 in the cancer stage group of HCC patients. (d) Box plot shows the relative expression of ABL1 in the TP53 mutation status group of HCC patients.

To evaluate the expression of ABL1 in HCC tissues and normal tissues, we used GEPIA to analyze 369 HCC specimens from TCGA. This analysis revealed that ABL1 expression was significantly higher in HCC tissues than in normal tissues (Figure 1). In addition, we performed a subgroup analysis based on age, sex, cancer stage, and TP53 mutation status (Figure 2) and found that ABL1 expression was significantly higher in HCC patients than in healthy people.

**Figure 3:**
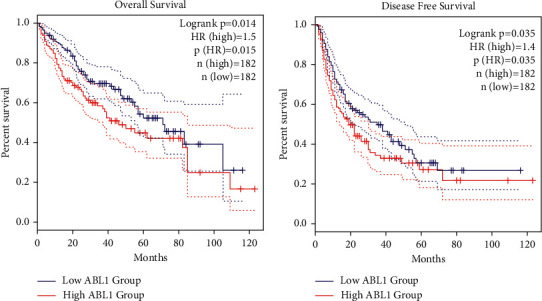
The prognostic value of ABL1 in HCC. (a) OS. (b) DFS.

**Figure 4:**
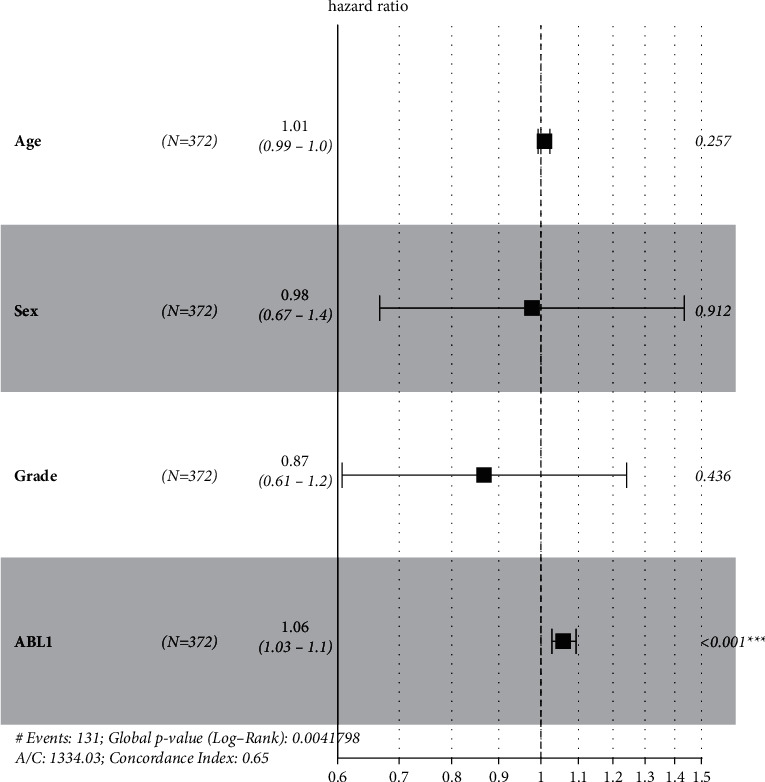
Multivariate analysis of ABL1 expression and other clinical characteristics.

In addition, we analyzed the association between ABL1 expression and clinicopathological characteristics in the TCGA HCC cohort (Table 1). ABL1 expression was significantly correlated with gender (<0.01), histological grade (*P*=0.013), and survival status (*P* < 0.01). We further analyzed the prognostic value of ABL1 in HCC. The survival curve from GEPIA showed that high expression of ABL1 predicts unfavorable OS (*P*=0.014) and DFS (*P*=0.035). The results are shown in Figure 3. We also explored the relationship between various clinicopathological features, ABL1 expression, and prognosis of HCC. The results of both univariate and multivariate analyses indicated that ABL1 expression was associated with the prognosis of HCC (Figure 4) (Table 2). These results suggest that ABL1 is a potentially effective independent prognostic marker for HCC.

### 3.2. ABL1-Related Functions and Pathways in HCC

**Figure 5:**
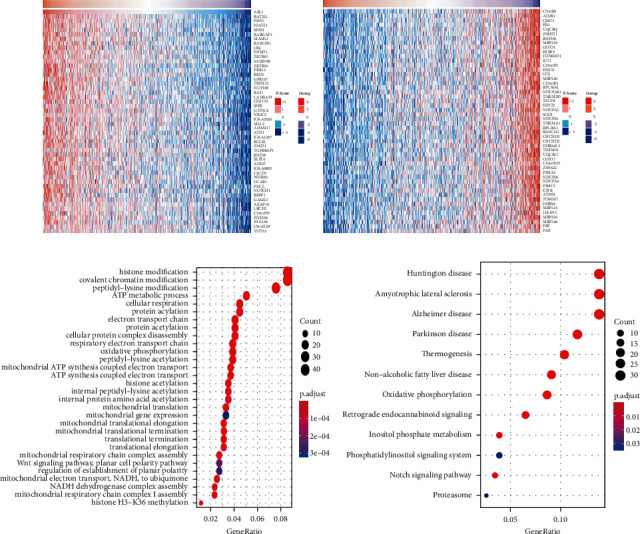
ABL1 coexpressed genes and functional enrichment. (a) The heat map shows the top 50 genes positively related to ABL1. (b) The heat map shows the top 50 genes negatively related to ABL1. (c) GO enrichment analysis. (d) KEGG enrichment analysis.

We explored the biological interaction network of ABL1 in HCC to clarify the biological function of ABL1. We first selected the genes related to ABL1 and performed an enrichment analysis. The top 50 genes with significant positive and negative correlations with ABL1 are shown in Figures 5(a) and 5(b). In addition, we performed GO and KEGG analyses. GO analysis showed that these genes mainly regulate mitochondrial activity, ATP metabolism, protein translation, and metabolism (Figure 5(c)). KEGG pathway analysis showed enrichment in various neurological diseases, nonalcoholic fatty liver disease, and the notch signaling pathway (Figure 5(d)). These findings indicated the potential role of ABL1 in HCC progression.

### 3.3. Association between ABL1 Expression and Tumor-Infiltrating Immune Cells

**Figure 6:**
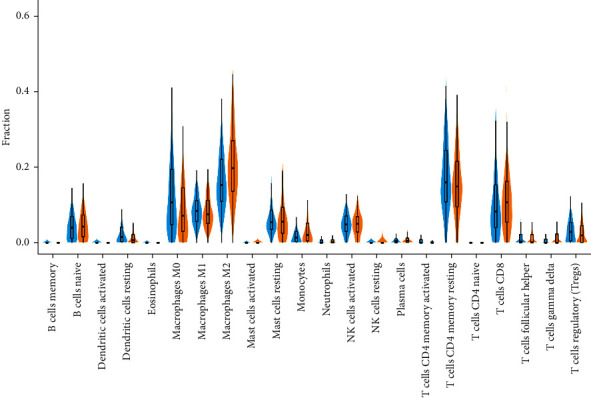
The ratios of 22 immune cell subpopulations are analyzed via CIBERSORT. Yellow represents high ABL1 expression group. Blue represents low ABL1 expression group.

Immune infiltration is closely related to tumor progression. Therefore, we also evaluated the effect of ABL1 expression on immune infiltrating cells in liver cancer using CIBERSORT. The proportions of 22 immune cell subgroups are shown in Figure 6. The results showed that B cells, dendritic cells, macrophages, mast cells, monocytes, NK cells, CD4 cells, and CD8 cells are significantly affected by ABL1 expression. Among the identified cells, naive B cells (*P*=0.001623), M2 macrophages (*P*=0.003429), mast cells resting (*P*=6.74*e* − 7), and NK cells resting (*P*=0.0002681) were most abundant in the high ABL1 expression group, whereas dendritic cells resting (*P*=4.747*e* − 05), M0 macrophages (*P*=3.308*e* − 12), CD4 cells (*P*=0.0008109), and CD8 cells (*P*=0.01354) were significantly reduced.

**Figure 7:**
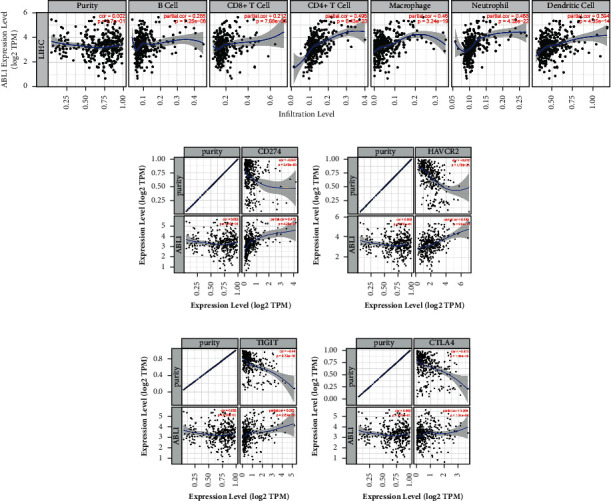
ABL1 expression affects immune cell infiltration in HCC. (a) The relationship between ABL1 expression and immune infiltrating cells was analyzed by TIMER. (b) ABL1 expression is positively correlated with PD-L1, TIGIT, TIM3, and CTLA4 expression.

We used TIMER to further study the association between ABL1 and the level of tumor immune cell infiltration (Figure 7(a)). The results revealed that high ABL1 expression was significantly positively correlated with B cells (*r* = 0.285, *P*=7.25*e* − 08), CD8 cells (*r* = 0.212, *P*=7.68*e* − 06), CD4 cells (*r* = 0.496, *P*=8.43*e* − 23), macrophages (*r* = 0.46, *P*=3.24*e* − 19), neutrophils (*r* = 0.488, *P*=4.32*e* − 22), and dendritic cells (*r* = 0.394, *P*=4.35*e* − 14) in HCC. These results suggest that ABL1 expression influences liver cancer progression by altering immune cell infiltration.

### 3.4. Association between ABL1 Expression and Immune Checkpoints

Immune checkpoint blockade therapy is a popular immunotherapy method and shows a strong therapeutic effect. We explored the relationship between ABL1 expression and tumor immunotherapy. We found that some immune checkpoints (PD-1L, TIM3, TIGIT, and CTLA4) were positively correlated with ABL1 expression (Figure 7(b)). In addition, these immune checkpoint markers were significantly expressed in the group with high ABL1 expression. We further explored the relationship between ABL1 and various infiltrating immune cell types. The results showed that the expression level of ABL1 was positively correlated with T cell exhaustion, T cells (general), CD8 + T cells, CD4 + T cells, Th1 cells, Th2 cells, Tfh cells, Th17 cells, Tregs, monocytes, TAMs, M1 macrophages, M2 macrophages, neutrophils, natural killer cells, and dendritic cells. The results are shown in Table 3. This finding suggests that ABL1 is involved in T cell exhaustion in the hepatocellular carcinoma tumor microenvironment.

## 4. Discussion

HCC can occur at any age and is most common in patients with chronic hepatitis [22]. Viral hepatitis B is highly prevalent in China, and HCC incidence shows an increasing trend year by year [23]. HCC is highly malignant, and surgical resection is currently the only curative therapeutic intervention. Although great progress has been made in developing diagnostic tools and treatments for liver cancer, the prognosis of most patients with advanced HCC is still very unsatisfactory. ABL1 is a gene that was first reported in leukemia. Subsequently, abnormal ABL1 expression was found in a variety of other tumors. However, there are few studies on the prognostic value and specific mechanism of ABL1 in liver cancer. Therefore, our research focused on the potential relationship between ABL1 and HCC. We aimed to evaluate the specific biological function of ABL1 in HCC through bioinformatics methods to identify ABL1-related pathways and determine the association between ABL1 and tumor immunity.

Our analysis showed that ABL1 expression was significantly higher in liver cancer tissues than in normal tissues. Subgroup analysis further confirmed that ABL1 mRNA was highly expressed in HCC. In addition, we used the GEPIA database to evaluate the prognostic value of ABL1 in HCC. We found that high ABL1 expression was significantly associated with adverse OS and DFS. Multivariate Cox regression analysis further showed that the ABL1 expression level was an independent risk factor for liver cancer prognosis.

Coexpressed genes act synergistically in strictly regulated biological processes, and thus they can provide alternative pathways to sidestep barriers, providing an adaptive evolution advantage [24]. We performed enrichment analysis of ABL1-related genes. GO analysis revealed some functional terms related to ABL1, such as the histone modification (*P*=2.48*e* − 09), the ATP metabolic process (*P*=2.27*e* − 05), and the Wnt signaling pathway (*P*=0.035) [25–27]. Functional enrichment analysis showed that ABL1 was associated with Huntington's disease (*P*=1.12*e* − 08), amyotrophic lateral sclerosis (*P*=3.0*e* − 07), Alzheimer's disease (*P*=3.59*e* − 07), and Parkinson's disease (*P*=6.53*e* − 08). Surprisingly, ABL1 was also associated with nonalcoholic fatty liver disease (*P*=5.96*e* − 08). In addition, we found pathways and metabolic processes related to tumors, such as oxidative phosphorylation (*P*=5.54*e* − 08), inositol phosphate metabolism (*P*=0.0035), and notch signaling pathway (*P*=0.0038). These results indicate that ABL1 is involved in a variety of diseases and may play an important role in them. The results also confirmed that ABL1 regulates the progression of HCC through a complex mechanism, revealing ABL1 as a potential target in HCC treatment.

HCC is a typical inflammatory-related tumor. Its tumor microenvironment includes a large number of immune cells, inflammatory factors, and extracellular matrix, forming a complex immune microenvironment. Immune cells in the tumor microenvironment of HCC mainly include tumor-infiltrating lymphocytes (TILs), tumor-associated macrophages (TAMs), tumor-associated neutrophils (TANs), myeloid-derived suppressor cells (MDSCs), and dendritic cells (DCs). TILs are composed of regulatory T cells, cytotoxic T lymphocytes, B cells, and NK cells in HCC. Regulatory T cells are a subset of CD4 + T cells, a group of lymphocytes with a high degree of immunosuppression, which can achieve immunosuppressive effects by inhibiting CD8 + T cells [28]. Studies displayed that regulatory T cells increased significantly in HCC and were related to tumor size, invasiveness, and prognosis [29, 30]. Lee et al. found that CD4 + CD25 + Treg infiltrated in HCC could effectively inhibit the immune response of dendritic cell [31]. CD8 + T cells are the main cytotoxic T lymphocytes that play an antitumor effect in HCC. Our previous study found that IL-21 produced by CD8 + T cells in HCC induced the differentiation of B cells into plasma cells, which stimulated humoral immunity and was associated with favorable prognosis [32]. In addition, the expression of Fas/FasL on CD8 + T cells was positively correlated with the antitumor immunity of liver cancer [33]. B cells can directly present tumor-related antigens to CD4 + T and CD8 + T cells to exert antitumor immunity or directly kill tumor cells. CD20 (+) B cells in the tumor microenvironment can produce IFN-*γ*, interleukin 12p40, granzyme B, and TRAIL and acted in cooperation with CD8 (+) T cells to promote tumor immunity and predict good prognosis in HCC [34]. However, a study found that PD-1 (hi) B cell infiltration in HCC could induce tumor immune tolerance and lead to poor prognosis [35]. NK cells are an important component of innate immunity, and their defects or inhibition of function can significantly affect the prognosis of HCC patients. Recently, some scholars have found that CD49a + NK cells, a subgroup of NK cells, highly expressing immune checkpoints PD-1 and TIGIT, exert immunosuppressive effects to promote poor prognosis in patients with HCC [36]. TAMs can be divided into M1 type and M2 type. M2 type macrophages can secrete a variety of cytokines and rely on a variety of ways to promote liver cancer invasion and metastasis [37]. However, M1 type can exert effective antitumor effects and inhibit the progression of liver cancer [38]. TANs also are multifaceted. On the one hand, it can recruit regulatory T cells and TAMs to promote liver cancer invasion and metastasis [39]. On the other hand, it can directly kill liver cancer cells and inhibit tumor progression [39]. MDSCs are a group of immature myeloid cells with strong immunosuppressive activity and can inhibit antitumor immunity from different ways. Studies revealed that targeting MDSC in HCC could enhance the antitumor effect of immune checkpoint inhibitors [40]. DCs are the most important antigen-presenting cells in the human body, which stimulate adaptive immune responses by presenting antigens to other immune cells. DCs can simultaneously regulate immune response and immune tolerance and play an important role in regulatory immunity. A report revealed that DCs changed from the early state of immune activation to the state of immunosuppression during the progression of liver cancer [41]. Another study showed that plasmacytoid dendritic cells infiltrated in HCC were a risk factor for poor prognosis [42]. They may induce the production of a variety of regulatory cells and inhibit the function of cytotoxic T cells leading to immune escape. We used CIBERSORT to analyze the tumor immune microenvironment of HCC. We found that ABL1 expression was significantly correlated with increased infiltration of B cells, dendritic cells, macrophages, CD4+ cells, and CD8+ cells. We further verified these results with TIMER and found that high ABL1 expression was positively correlated with B cells (*r* = 0.285, *P*=7.25*e* − 08), CD8 cells (*r* = 0.212, *P*=7.68*e* − 06), CD4+ cells (*r* = 0.496, *P*=8.43*e* − 23), macrophages (*r* = 0.46, *P*=3.24*e* − 19), neutrophils (*r* = 0.488, *P*=4.32*e* − 22), and dendritic cells (*r* = 0.394, *P*=4.35*e* − 14) in HCC. Among these immune cells, CD4+ cells were most strongly associated with ABL1 expression. The findings indicate that ABL1 has an important effect on immune infiltrating cells in HCC.

Immune checkpoints have been proven to be effective targets for the treatment of tumors. Studies have shown that the expression of immune checkpoints such as PD-1L, TIM3, TIGIT, and CTLA4 can affect tumor progression and thus change patient prognosis [43–46]. Immunosuppressants based on immune checkpoints have been effectively used in clinical practice. In the current study, we found that ABL1 expression was positively correlated with PD-1L, TIM3, TIGIT, and CTLA4 expression. Interestingly, we also found that high expression of ABL1 was positively correlated with the expression of markers of these immune checkpoints, such as STAT1, STAT3, STAT4, STAT6, STAT5A, and BCL6. In addition, we also observed that ABL1 expression was significantly correlated with monocytes, tumor-associated macrophages, M1/M2 macrophages, NK cells, and dendritic cells. These results show that ABL1 can regulate immune cell infiltration and affect the progression of HCC.

## 5. Conclusion

Our results demonstrated that high ABL1 expression is associated with unfavorable prognosis in HCC. The high ABL1 expression significantly influences the immune cell infiltration and immune checkpoint expression in the tumor microenvironment in HCC. We hypothesize that specific molecular targeting ABL1 expression could affect immune cell infiltration in the tumor microenvironment and improve the prognosis of patients with HCC. Targeting ABL1 expression may effectively strengthen the effectiveness of other immune checkpoint inhibitors in HCC. ABL1 may be a promising prognostic biomarker and therapeutic target for HCC patients. Our research provides a basis for the role of ABL1 in HCC, and further research is strongly recommended.

## Figures and Tables

**Table 1:** Association between ABL1 expression and clinicopathological characteristics in the TCGA HCC cohort.

Variable	Low ABL1 expression (*n* = 170)	High ABL1 expression (*n* = 171)	*X* ^2^	*P* value
Age			0.8026	0.3703
≤50	33	40		
>50	137	131		

Gender			6.6817	<0.01
Female	49	80		
Male	121	111		

TNM stage			3.1116	0.119
I/II	124	114		
III/IV	46	57		

Histologic grade			6.1729	0.013
G1/G2	123	91		
G3/G4	47	80		

Survival status				
Alive	113	105	48.0403	<0.01
Dead	57	66		

**Table 2:** Univariate and multivariate Cox regression of ABL1 expression for overall survival in patients with HCC.

Variable	Univariate Cox regression HR (95% CI)	*P* value	Multivariate Cox regression HR (95% CI)	*P* value
Age	1.008 (0.995–1.022)	0.221	1.0083 (0.9940–1.023)	0.257303
Sex	0.8039 (0.5647–1.144)	0.226	0.9787 (0.6671–1.436)	0.912241
Grade	0.8703 (0.6133–1.235)	0.437	0.8675 (0.6068–1.240)	0.435503
ABL1	1.062 (1.032–1.093)	4.61*E* − 05	1.0597 (1.0286–1.092)	0.000138

**Table 3:** Spearman correlation analysis between ABL1 expression and markers of immune cells in HCC.

Terms	Markers	R	*P* value
T cell exhaustion	PDCD1 (PD-1)	0.2	^*∗∗∗*^
	CTLA4	0.111	^*∗*^
	LAG3	0.063	0.198
	HAVCR2 (TIM3)	0.281	^*∗∗∗*^
	GZMB	0.086	0.078
	BTLA	0.087	0.074
	CD244 (SLAMF4)	0.041	0.403
	CD274 (PD-L1)	0.201	^*∗∗∗*^
	CD96	0.201	^*∗∗∗*^
	IDO1	0.1	^*∗*^
	KDR	0.162	^*∗∗∗*^
	PDCD1LG2 (PD-L2)	0.197	^*∗∗∗*^
	TGFBR1	0.498	^*∗∗∗*^
	TIGIT	0.145	^*∗∗*^

T cell (general)	CD3E	0.145	^*∗∗*^
	CD3G	0.197	^*∗∗∗*^
	CD28	0.181	^*∗∗∗*^
	CD2	0.137	^*∗∗*^

CD8 + T cells	CD8A	0.144	^*∗∗*^
	CD8B	0.098	^*∗*^

CD4 + T cells	CD4	0.059	0.228
	CD40LG (CD40L)	0.143	^*∗∗*^
	CXCR4	0.306	^*∗∗∗*^

Th1 cells	TBX21	0.093	0.055
	STAT4	0.242	^*∗∗∗*^
	STAT1	0.211	^*∗∗∗*^
	IFNG	0.082	0.093

Th2 cells	STAT6	0.34	^*∗∗∗*^
	STAT5A	0.448	^*∗∗∗*^

Tfh cells	BCL6	0.306	^*∗∗∗*^
	IL-21	0.092	0.058

Th17 cells	STAT3	0.306	^*∗∗∗*^
	IL17A	0.092	0.058

Treg	FOXP3	−0.035	0.473
	STAT5B	0.375	^*∗∗∗*^
	TGFB1	0.342	^*∗∗∗*^
	IL2RA (CD25)	0.271	^*∗∗∗*^

B cell	CD19	0.072	0.141
	CD79A	0.058	0.235

Monocyte	CD86 (B7-2)	0.251	^*∗∗∗*^
	CSF1R	0.281	^*∗∗∗*^

TAM	CCL2	0.176	^*∗∗∗*^
	CD68	0.014	0.777
	IL10	0.258	^*∗∗∗*^

M1 macrophage	IRF5	0.316	^*∗∗∗*^
	PTGS2	0.142	^*∗∗*^

M2 macrophage	CD163	0.09	0.063
	VSIG4	0.164	^*∗∗∗*^
	MS4A4A	0.219	^*∗∗∗*^

Neutrophils	CEACAM8	0.039	0.428
	ITGAM	0.363	^*∗∗∗*^
	CCR7	0.088	0.071

Natural killer cell	FCGR3A (CD16)	0.164	^*∗∗∗*^
	NCAM1 (CD56)	0.211	^*∗∗∗*^
	KIR2DL1	0.067	0.167
	KIR2DL3	0.157	^*∗∗*^
	KIR2DL4	0.171	^*∗∗∗*^
	KIR3DL1	0.104	^*∗*^
	KIR3DL2	0.13	^*∗∗*^
	KIR2DS4	0.01	0.844

Dendritic cell	HLA-DRA	0.148	^*∗∗*^
	HLA-DPA1	0.144	^*∗∗*^
	CD1C	0.23	^*∗∗∗*^
	NRP1	0.53	^*∗∗∗*^
	ITGAX	0.261	^*∗∗∗*^

TAM, tumor-associated macrophages; Tfh cells, T follicular helper cells; Treg, T regulatory cells. ^*∗*^*P* < 0.05, ^*∗∗*^*P* < 0.01, and ^*∗∗∗*^*P* < 0.001.

## Data Availability

All data can be obtained from the first author or corresponding author.

## Abbreviations

HCC: Hepatocellular carcinoma
ABL1: Abelson tyrosine-protein kinase 1
OS: Overall survival
DFS: Disease-free survival
GEPIA: Gene Expression Profiling Interactive Analysis
KEGG: Kyoto Encyclopedia of Genes and Genomes
TCGA: The Cancer Genome Atlas
TILs: Tumor-infiltrating lymphocytes
TAMs: Tumor-associated macrophages
TANs: Tumor-associated neutrophils
MDSCs: Myeloid-derived suppressor cells
DCs: Dendritic cells.
